# Chronic Tear Deficiency Sensitizes Transient Receptor Potential Vanilloid 1-Mediated Responses in Corneal Sensory Nerves

**DOI:** 10.3389/fncel.2020.598678

**Published:** 2020-12-23

**Authors:** Takayoshi Masuoka, Yuka Yamashita, Katsuya Nakano, Kenshi Takechi, Takahiro Niimura, Masashi Tawa, Qiang He, Keisuke Ishizawa, Takaharu Ishibashi

**Affiliations:** ^1^Department of Pharmacology, School of Medicine, Kanazawa Medical University, Uchinada, Japan; ^2^Department of Clinical Pharmacology and Therapeutics, Institute of Biomedical Sciences, Tokushima University Graduate School, Tokushima, Japan; ^3^Department of Drug Information Analysis, College of Pharmaceutical Sciences, Matsuyama University, Matsuyama, Japan

**Keywords:** cornea, transient receptor potential vanilloid 1 (TRPV1), trigeminal ganglion, lacrimal gland excision, cold-sensitive nerve

## Abstract

Chronic tear deficiency enhances the excitability of corneal cold-sensitive nerves that detect ocular dryness, which can lead to discomfort in patients with dry eye disease (DED). However, changes in corneal nerve excitations through the polymodal nociceptor “transient receptor potential vanilloid 1” (TRPV1) and the potential link between this receptor and symptoms of DED remain unclear. In this study, we examined the firing properties of corneal cold-sensitive nerves expressing TRPV1 and possible contributions of chronic tear deficiency to corneal nerve excitability by TRPV1 activation. The bilateral excision of lacrimal glands in guinea pigs decreased the tear volume and increased the frequency of spontaneous eyeblinks 1–4 weeks after surgery. An analysis of the firing properties of the cold-sensitive nerves was performed by single-unit recordings of corneal preparations 4 weeks after surgery in both the sham-operated and gland-excised groups. Perfusion of the TRPV1 agonist, capsaicin (1 μM), transiently increased the firing frequency in approximately 46–48% of the cold-sensitive nerves characterized by low-background activity and high threshold (LB-HT) cold thermoreceptors in both groups. Gland excision significantly decreased the latency of capsaicin-induced firing in cold-sensitive nerves; however, its magnitude was unchanged. Calcium imaging of cultured trigeminal ganglion neurons from both groups showed that intracellular calcium elevation of corneal neurons induced by a low concentration of capsaicin (0.03 μM) was significantly larger in the gland excision group, regardless of responsiveness to cold. An immunohistochemical study of the trigeminal ganglion revealed that gland excision significantly increased the proportion of corneal neurons enclosed by glial fibrillary acidic protein (GFAP)-immunopositive satellite glial cells. Topical application of the TRPV1 antagonist, A784168 (30 μM), on the ocular surface attenuated eye-blink frequency after gland excision. Furthermore, gland excision enhanced blink behavior induced by a low concentration of capsaicin (0.1 μM). These results suggest that chronic tear deficiency sensitizes the TRPV1-mediated response in the corneal LB-HT cold thermoreceptors and cold-insensitive polymodal nociceptors, which may be linked to dry eye discomfort and hyperalgesia resulting from nociceptive stimuli in aqueous-deficient dry eyes.

## Introduction

Dry eye disease (DED) is defined as a multifactorial disease of tears and ocular surface and is characterized by symptoms of dry eye discomfort, visual disturbances, and tear film instability, with potential damage to the ocular surface (Nelson et al., 2017). Hundreds of millions of people worldwide are affected by DED and experience severe dry eye symptoms and pain that significantly impact their quality of life. Therefore, it is crucial that the treatment of DED target these symptoms; however, the underlying mechanisms and effective treatments of this disease are not well established.

Sensory nerve terminals, projected from the trigeminal ganglion, are distributed throughout the corneal surface and detect nociceptive and non-nociceptive somatosensory information on the ocular surface. Corneal nerve terminals equipped with transient receptor potential melastatin 8 (TRPM8), a cold sensor, are susceptible to temperature decreases and elevation of osmolarity due to the evaporation of tear film (Parra et al., 2010; Belmonte and Gallar, 2011). Activation of these cold-sensitive nerves is associated with a sensation of dryness and basal tear secretion to avoid desiccation of the ocular surface. Recent studies have revealed that some animal models of the ocular disease show abnormal firing responses in the corneal cold-sensitive nerves. Allergic keratoconjunctivitis and ultraviolet keratitis have been found to decrease the basal firing activities of corneal cold-sensitive nerves and its response to cooling (Acosta et al., 2013, 2014). Chronic ocular dryness in experimental dry eye models exhibits neuropathic firing in corneal TRPM8-positive nerves, which may be associated with dry eye discomfort in DED (Kovács et al., 2016). Aging, a risk factor for dry eye symptoms, may be associated with a change in the morphology and firing patterns of the corneal cold-sensitive nerves with TRPM8 (Alcalde et al., 2018). Hence, the dynamics of cold-sensitive nerve activities may be the key underlying mechanism of ocular dryness and discomfort in ocular surface disease.

Transient receptor potential vanilloid 1 (TRPV1), a polymodal nociceptor activated by acidic solutions, inflammatory lipids, and pungent compounds, is predominantly localized in the sensory neurons with C and Aδ fibers. Sensitization of the TRPV1-mediated response in the primary sensory neurons plays a pivotal role in the development of inflammatory and neuropathic pain and is thought to be a therapeutic target for chronic pain disorders (Fischer et al., 2013; Shutov et al., 2016). Corneal TRPV1 is involved in the maintenance of the corneal structure, re-epithelialization, and inflammation in corneal injury (Okada et al., 2015). For example, the corneal TRPV1 signal in epithelial debridement is associated with healing of the epithelium, possibly through the upregulation of interleukin-6 (IL-6) and substance P in mice (Sumioka et al., 2014). Loss of the *Trpv1* gene was found to delay healing of stromal incision injuries through the transforming growth factor β1 (TGFβ1)-induced formation of granulation tissue in the corneas of mice (Nidegawa-Saitoh et al., 2018). In addition to the restorative effects of TRPV1 in the cornea, TRPV1 in the corneal sensory nerves may be responsible for burning and stinging pain, since topical treatment with the potent TRPV1 agonist, resiniferatoxin, deletes peptidergic sensory nerves, leading to a reduction of capsaicin-induced nociceptive responses (Bates et al., 2010). In a previous report, keratoconjunctivitis was reported to augment the firing of polymodal stimuli in ciliary fibers that innervate the cornea (Acosta et al., 2013). Also, blinking behavior in guinea pigs related to ocular discomfort is reversed by treatment with the TRPV1 blocker, capsazepine (Acosta et al., 2013). Recently, a novel siRNA for TRPV1, tivanisiran, was designed to reduce ocular pain and was shown to improve ocular hyperemia and tear quality in dry-eye in human and animal models (Moreno-Montañés et al., 2018). Therefore, corneal TRPV1 may be important for healing corneal tissue, tear secretion, and alleviating the pain in inflammatory disorders of the ocular surface.

Clinical and basic research has indicated that chronic inflammation of the ocular surface underlies the pathology of dry eye (Craig et al., 2017); hence, we hypothesized that chronic ocular dryness causes abnormalities in sensory nerve excitations through the nociceptor TRPV1 in the same manner as in other inflammatory disorders. A morphological study reported that 6% of TRPV1-positive neurons co-express TRPM8, except the mechanosensor Piezo2 in corneal afferent neurons (Alamri et al., 2015), indicating that some corneal cold-sensitive nerves express TRPV1 channels and have the ability to respond to endogenous and exogenous nociceptive stimuli. Therefore, we first examined the firing properties of cold-sensitive nerves with functional corneal TRPV1. Subsequently, to clarify the contribution of TRPV1 to the discomfort localized to the ocular surface in DED, changes in the TRPV1-mediated response in the corneal cold-sensitive and cold-insensitive neurons and blink behavior were investigated through lacrimal gland excision in guinea pigs that chronically lacked tear secretion.

## Materials and Methods

### Animals

Four-week-old male Hartley guinea pigs were purchased from SLC (Shizuoka, Japan) and housed in cages in a temperature-controlled room (25 ± 1°C), with a 12-h light/dark cycle (light on from 7:00 to 19:00). All animal procedures were approved by the Ethics Committee of Kanazawa Medical University (2016-55, 2017-55), and the animals were treated humanely following the “Guiding Principles for the Care and Use of Laboratory Animals” established by the Japanese Pharmacological Society.

### Surgery

Guinea pigs with excised lacrimal glands were used as an aqueous-deficit dry eye model based on a previous report (Masuoka et al., 2018). The bilateral lacrimal glands were carefully removed from guinea pigs anesthetized with ketamine (90 mg/kg, i.p., Daiichi Sankyo, Tokyo, Japan) and xylazine (5 mg/kg, i.p., Zenoaq, Fukushima, Japan). The incision was disinfected with 0.3% tobramycin (Nitto Medic Company, Toyama, Japan) and sutured. The sham-operated animals were treated by performing a skin incision, adding a drop of the antibiotic in the surgical area without gland excision, and subsequent suturing of the incision. Animals received water and food *ad libitum* and were bred in individual cages postoperatively for 4–5 weeks.

### Measurement of Eye-Blink Behavior and Tear Volume

Blink behavior and tear volume were measured before and 1–4 weeks after surgery in the sham-operated and gland-excised guinea pigs. These experiments were conducted based on procedures described in a previous report (Acosta et al., 2013). The researchers that performed these experiments were blinded to the experimental group to avoid subjective bias. Spontaneous blink frequency was assessed by the number of blinks registered for each eye, which were counted twice for 5 min by two different observers while the animal was freely moving in a clear acryl cage (80 × 60 × 60 cm). After the blinks had been counted, the volume of tear fluid was measured in both eyes using phenol red threads (Zone-Quick; AYUMI Pharmaceutical Company, Tokyo, Japan). The lower lid was pulled down slightly, and the folded 2-mm end of the thread was gently placed onto the nasal palpebral conjunctiva. The thread was removed after 30 s, and the length of the red-stained portion was measured with an accuracy of ± 0.1 mm using Vernier calipers to determine the tear volume in the conjunctival sac and the tear secretion throughout this period. To assess blink behavior after applying eye drops, a 10-μl drug solution dissolved in 0.9% sodium chloride (saline) containing 0.1% ethanol was topically administered to the ocular surface. Saline containing 0.1% ethanol (vehicle) was prepared to serve as a control for the drug solution. The number of blinks was counted for 2 or 5 min while the animals were in a cage.

### Single-Unit Recording in Acute Corneal Preparations

Single-unit recording of the corneas was conducted based on a previous report (Masuoka et al., 2018). Four weeks after surgery, the guinea pigs were anesthetized with ketamine and xylazine, and the corneas were excised with a circular cut around the limbus. The corneas were incubated in Krebs–Henseleit solution (138.6 mM NaCl, 3.35 mM KCl, 21 mM NaHCO_3_, 9.9 mM glucose, 0.6 mM NaH_2_PO_4_, 2.5 mM CaCl_2_, and 1 mM MgCl_2_, gassed with a mixture of 95% O_2_ and 5% CO_2_, pH 7.4) for more than 1 h and then pinned to the bottom of a recording chamber. The Krebs–Henseleit solution maintained at 34°C was continuously perfused (4 ml/min) with a feedback-controlled Peltier device (CL-100 and SC-20, Warner Instruments, Hamden, CT, USA), and the temperature was monitored using a thermometer (BAT-12 and IT-18, Physitemp, Clifton, NJ, USA). Nerve terminal impulse (NTI) activity was recorded using a glass micropipette applied to the corneal surface with a micromanipulator and fixed by slight suction. Pipettes with a tip diameter of approximately 50 μm were filled with the bath solution. Signals were recorded with respect to an Ag/AgCl pellet (World Precision Instruments, Sarasota, FL, USA) in the bath. Electrical activity was amplified with an AC/DC differential amplifier (model 3000, A-M System Inc., Sequim, WA, USA; high-pass 100 Hz, low-pass 5 kHz). The data were transferred to a personal computer with a CED micro1401 acquisition system (Cambridge Electronic Design Limited, Cambridge, UK) and analyzed with Spike2 software (Cambridge Electronic Design Limited, Cambridge, UK). NTI was explored on the corneal surface with the tip of the micropipette until the spontaneous activity of a single nerve terminal was detected at a specific site. After stable NTI activities were obtained at the basal temperature of 34°C, cold stimulation was applied. When the temperature of the bath solution was reduced to 20°C (at approximately −0.4°C/s), most of the NTI frequencies markedly increased. We defined cold-sensitive nerves as those in which the maximal NTI frequency during cooling was more than double that of the averaged NTI frequency at the basal temperature. The following three parameters were calculated to characterize each cold-sensitive nerve: (1) spontaneous NTI frequency at the basal temperature (ongoing activity); (2) the decrease in temperature required to increase NTI frequency by 50% of the ongoing activity during cooling ramp (cooling threshold); and (3) the maximal NTI frequency during cooling (cooling response). At more than 5 min after return to basal temperature, 1 μM capsaicin was perfused into the chamber for 2 min at 34°C. Capsaicin-sensitive nerves were defined as those in which the NTI frequency during perfusion transiently increased to more than double. The sensitivity and magnitude of capsaicin-induced responses were assessed based on the response latency time until the NTI frequency increased by more than 1.5 times the ongoing activity and the total NTI activities during perfusion, respectively.

### Primary Culture of the Corneal Trigeminal Ganglion Neurons

The trigeminal ganglion neurons innervating the corneas were retrogradely labeled under anesthesia with ketamine and xylazine in guinea pigs 4 weeks after surgery, as previously described (Kovács et al., 2016). Pieces of Spongel® film (6 mm diameter, 0.5 mm thickness; Astellas Pharma Inc., Tokyo, Japan) were saturated with 5 mM of FM1-43 (SynaptoGreen^TM^ C4, Biotium, Fremont, CA, USA) in saline and were carefully placed on the center of both corneas. After 40 min, the film was removed, and the eyes were repeatedly washed with saline. Four days after labeling, the guinea pigs were decapitated under anesthesia, and the first branches (V1) of the bilateral trigeminal ganglia were rapidly dissected in Ca^2+^/Mg^2+^-free Krebs–Henseleit solution (143.9 mM NaCl, 3.35 mM KCl, 21 mM NaHCO_3_, 9.9 mM glucose, 0.6 mM NaH_2_PO_4_) and gassed with a mixture of 95% O_2_ and 5% CO_2_ (pH 7.4). The trigeminal ganglion neurons were dissociated following treatment with collagenase XI (0.66 mg/ml, Sigma–Aldrich, St. Louis, MO, USA) and dispase II (3 mg/ml, Fujifilm, Tokyo, Japan) in a Ca^2+^/Mg^2+^-free solution and were shaken in a water bath at 37°C for 1 h. The cells were washed with a Ca^2+^/Mg^2+^-free solution, gently triturated, and passed through a 100-μm cell strainer (BD Biosciences, San Jose, CA, USA). The cell suspension was then layered on top of a 14% Percoll (GE Healthcare Life Sciences, Pittsburgh, PA, USA) gradient and centrifuged at 2,500 rpm for 13 min at room temperature. The pellet was suspended in culture medium [Dulbecco’s Modified Eagle Medium (Sigma–Aldrich) containing 10% horse serum (Gibco®, Thermo Fisher Scientific, Waltham, MA, USA), 5% fetal calf serum (Gibco®), and 1% penicillin-streptomycin (Wako, Osaka, Japan)]. The dispersed cells were seeded on glass coverslips coated with poly-L-lysine (Matsunami Glass Ind., Osaka, Japan). The medium was added 2 h after seeding, and calcium imaging experiments were performed 24–48 h after dissociation.

### Calcium Imaging

Changes in intracellular calcium were measured with the fluorescent calcium indicator, Fura-2, as previously described (Masuoka et al., 2015, 2017). For the microscopic fluorometric measurement, cultured trigeminal ganglion neurons were washed twice with Krebs–Henseleit solution and incubated for 1 h in a solution containing 3 μM of Fura-2-acetoxymethyl ester (Fura-2 AM; Dojindo Laboratories, Kumamoto, Japan) and 0.005% Cremophor EL (Sigma–Aldrich). After incubation, the cells were washed in Krebs–Henseleit solution for 30 min, and culture dishes were placed on the stage of an inverted microscope (ECLIPSE TE 300, Nikon, Tokyo, Japan) equipped with a 20× S-fluor objective. Fluorescence images were recorded and analyzed using a video image analysis system (HCimage, Hamamatsu Photonics, Hamamatsu, Japan). The experimental agents were dissolved in Krebs–Henseleit solution and delivered by continuous perfusion in the recording chamber with a peristaltic pump (2 ml/min). The perfused solutions were maintained at 34°C with a temperature controller (TC-344C and SH-27B, Warner Instruments). Image pairs of Fura-2 fluorescence were captured every 10 s at an emission wavelength of 510 nm by exciting Fura-2 at 340 and 380 nm. The 340–380 nm fluorescence ratio (F340/F380) was used as a parameter of intracellular calcium concentration.

### Immunohistochemistry of Trigeminal Ganglion

Four weeks after surgery, the corneal neurons were retrogradely labeled with Spongel® film saturated with 5 mM FM 1-43FX (Thermo Fisher Scientific, Waltham, MA, USA), in the same manner as described in the primary cultures of the corneal trigeminal ganglion neurons. Four days later, the guinea pigs under anesthesia were transcardially perfused with cold Krebs–Henseleit solution, followed by a cold fixative solution containing 4% paraformaldehyde in 0.1 M phosphate buffer (pH 7.4). After perfusion, the trigeminal ganglia were dissected and immersed in the same fixative for more than 24 h at 4°C. These ganglia were then placed in 0.01 M phosphate-buffered saline (PBS) containing 20% sucrose (w/v) for 12 h for cryoprotection. The specimens were embedded in Tissue Tek (Sakura Finetek, Torrance, CA, USA), and 10-μm sections were cut in the horizontal plane along the long axis of the ganglion. Subsequently, the sections were incubated with mouse anti-glial fibrillary acidic protein (GFAP) polyclonal antibody (1:1,000, GeneTex, #GTX40996, Irvine, CA, USA) diluted with 0.01 M PBS containing 4% normal goat serum and 0.3% Triton X 100 for 24 h at 4°C. After washing with 0.01 M of PBS for 30 min, the sections were incubated with Cy5 anti-mouse IgG heavy and light chains (1:1,000, Abcam, #ab6563, Cambridge, UK) for 2 h at room temperature. The sections were coverslipped in a mounting medium after rinsing with 0.01 M PBS and were examined under a fluorescence microscope (BZ-9000 system, Keyence, Osaka, Japan).

### Drugs

Capsaicin was obtained from Sigma–Aldrich; 3,6-Dihydro-3′-(trifluoromethyl)-*N*-[4-[(trifluoromethyl)sulfonyl]phenyl]-[1(2*H*), 2′-bipyridine]-4-carboxamide (A784168) was obtained from Tocris Bioscience (Bristol, UK). All drugs were dissolved in ethanol (stock solution), which was diluted in saline or Krebs–Henseleit solution immediately before the experiments. The osmolality of all solutions applied to cultured neurons, corneal tissue, and the ocular surface was adjusted to 305–315 mOsm/L with an osmometer (OSMOMAT3000, Gonotec, Berlin, Germany).

### Statistical Analyses

Data were analyzed using SigmaPlot version 14.0 software (Systat Software Inc., San Jose, CA, USA). The results are expressed as mean ± standard error of the mean (SEM). A two-way analysis of variance (ANOVA) or unpaired Student’s *t-*test was used. If a significant difference was found by two-way ANOVA, Dunnett’s *post hoc* test was conducted for the groups. Chi-squared tests were used to assess changes in the proportion of nerves or neurons.

## Results

### Lacrimal Gland Excision Decreases Tear Volume and Augment Blink Behavior in Guinea Pigs

**Figure 1:**
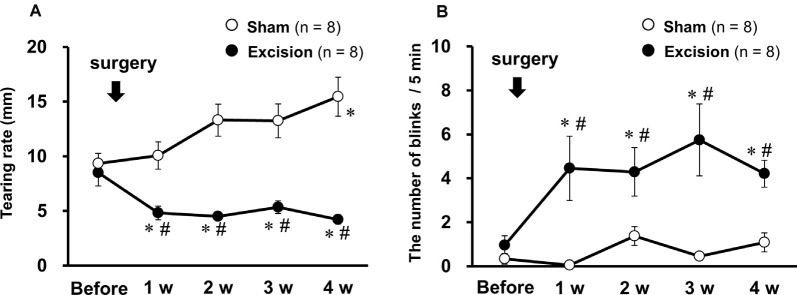
Changes in tear volume and spontaneous eye-blink behavior after bilateral excision of the lacrimal glands. **(A)** Tear volume is expressed as the mean wetted length of the phenol red threads of both eyes in the sham-operated (open circles) and gland excision groups (closed circles). **(B)** The average number of blinks in both eyes within 5 min in both groups. Each point and the corresponding vertical bar represent the mean ± SEM. *P < 0.05 vs. before surgery, ^#^*P* < 0.05 vs. the sham-operated group [two-way analysis of variance (ANOVA) followed by Dunnett’s test].

The effects of lacrimal gland excision on tear volume and blink behavior were examined in guinea pigs after surgical resection of the bilateral lacrimal glands or sham operation. Tear volume, measured by phenol red thread, substantially decreased 1–4 weeks after gland excision, as in a previous study (Kovács et al., 2016), but gradually increased in the sham-operated group (Figure 1A). The number of spontaneous blinks recorded for 5 min significantly increased 1–4 weeks after surgery in the excision group, but not in the sham-operated group (Figure 1B). These results indicate that the removal of the lacrimal glands induces chronic tear deficiency and simultaneously enhances blink frequency, which could reflect ocular discomfort and pain.

### Cold-Sensitive Nerves Expressing TRPV1 Channels Are Characterized by Low-Background Activity and a High Threshold to Cooling Stimuli

**Figure 2:**
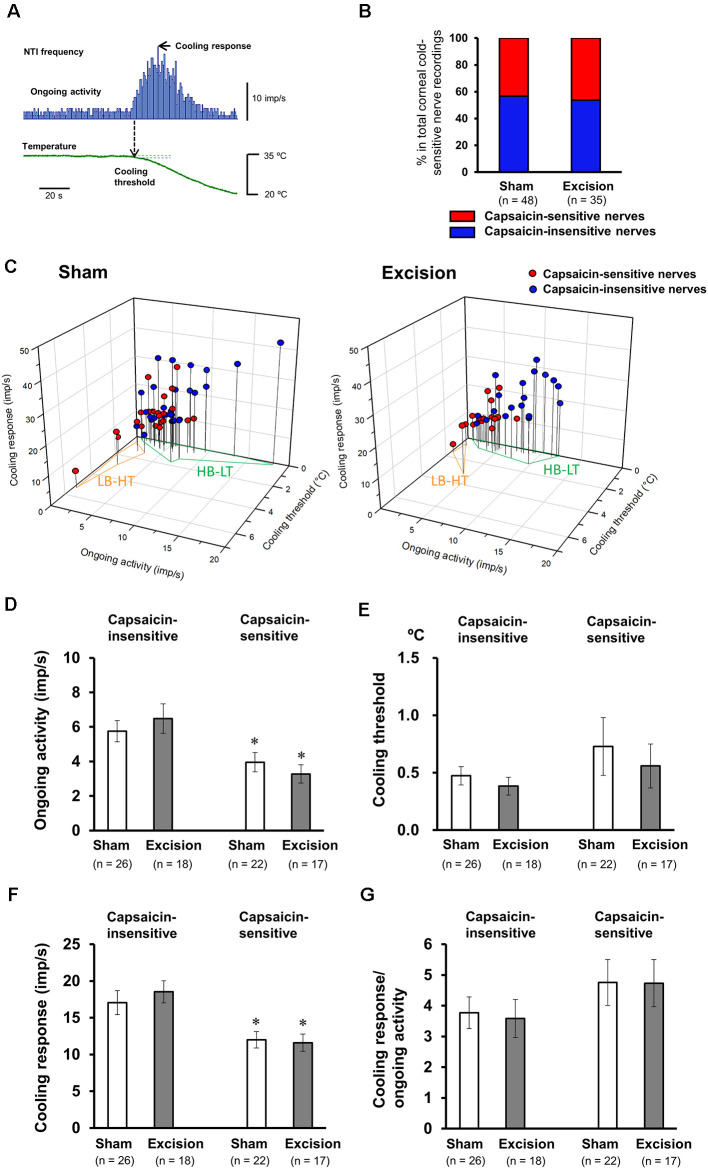
Firing characteristics of the cold-sensitive nerves responsive to capsaicin stimuli. **(A)** Representative nerve terminal impulse (NTI) frequency before and during cooling stimulation. **(B)** The proportion of nerve terminals responsive to capsaicin in the total recordings of the cold-sensitive nerves in the sham-operated and gland excision groups. **(C)** Individual recordings of the cold-sensitive nerves in the cornea dissociated from the sham-operated (left panel) and gland excision groups (right panel) plotted according to ongoing activity (*x*-axis), cooling response (*y*-axis), and cooling threshold (*z*-axis). The blue and red plots represent the capsaicin-insensitive and capsaicin-sensitive nerves, respectively. Areas enclosed in the orange line and green line correspond to the low-background activity-high threshold (LB-HT) and high background activity-low threshold (HB-LT) cold thermoreceptors, respectively. **(D–G)** Average values of the ongoing activity **(D)** cooling threshold **(E)**, cooling response **(F)**, and the ratio of cooling response to ongoing activity **(G)**. Each column and vertical bar represent the mean ± SEM. *P < 0.05 vs. capsaicin-insensitive group (two-way ANOVA followed by Dunnett’s test).

**Figure 3:**
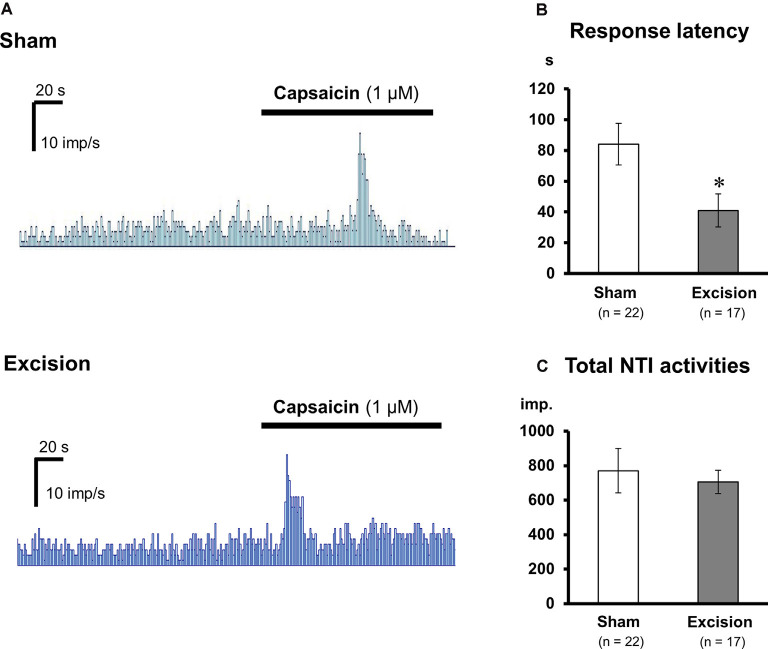
Changes in the capsaicin-induced response of corneal cold-sensitive nerves after lacrimal gland excision. **(A)** Representative NTI frequency of the corneal cold-sensitive nerves in the sham-operated (upper) and gland excision groups (bottom). **(B)** Response latency to capsaicin stimuli. **(C)** The total number of NTIs during capsaicin perfusion. Each column and vertical bar represent the mean ± SEM. *P < 0.05 vs. the sham-operated group (Student’s *t*-test).

We recorded NTI activity of corneal preparations stimulated by cooling and using a TRPV1 agonist, capsaicin, to examine the firing properties of corneal cold-sensitive nerves with functional TRPV1. Upon cooling the Krebs–Henseleit solution in the recording chamber to 20°C with a Peltier device, the NTI frequency in cold-sensitive nerves markedly increased (Figure 2A), as previously described (Masuoka et al., 2018). At more than 5 min after return to basal temperature (34°C), 1 μM capsaicin was perfused into the recording chamber. In 46% and 48% of corneal cold-sensitive nerves, NTI frequency transiently increased during perfusion in the sham-operated and gland excision groups, respectively (Figures 2B, Figure 3A). Subsequently, three parameters of each cold-sensitive nerve activity (ongoing activity, cooling threshold, and cooling response; 2A) were calculated and compared between capsaicin-sensitive and capsaicin-insensitive nerves. As shown in Figure 2C, all recordings were plotted on 3D graphs, and the capsaicin-sensitive nerves (red circles) tended to have lower background (ongoing) activities and higher thresholds for cooling than capsaicin-insensitive ones (blue circles) in both the sham-operated and gland excision groups. The average values of the three parameters are shown in Figures 2D–F. The capsaicin-sensitive nerve terminals showed significantly low ongoing activities (Figure 2D) and small cooling responses (Figure 2F) and tended to have high thresholds for cooling (Figure 2E) in both groups. When the cooling response was normalized with ongoing activity in each recording (Figure 2G), there was no significant difference between capsaicin-sensitive and capsaicin-insensitive nerves, suggesting that the reduced cooling responses of capsaicin-sensitive nerves may be caused by a reduction in ongoing activities. Lacrimal gland excision had no significant effect on any parameters in capsaicin-sensitive and capsaicin-insensitive nerves (Figures 2D–G), although gland excision appeared to be associated with increased cold response in previous studies (Kovács et al., 2016). In summary, functional TRPV1 in corneal cold-sensitive nerves may be predominantly located in low-background activity and high threshold (LB-HT) cold thermoreceptors. Chronic tear deficiency had no significant effect on sensitivity to cold in TRPV1-positive and TRPV1-negative cold-sensitive nerves.

### TRPV1-Mediated Responses Are Sensitized in Cold-Sensitive Nerves of Tear-Deficient Corneas

**Figure 4:**
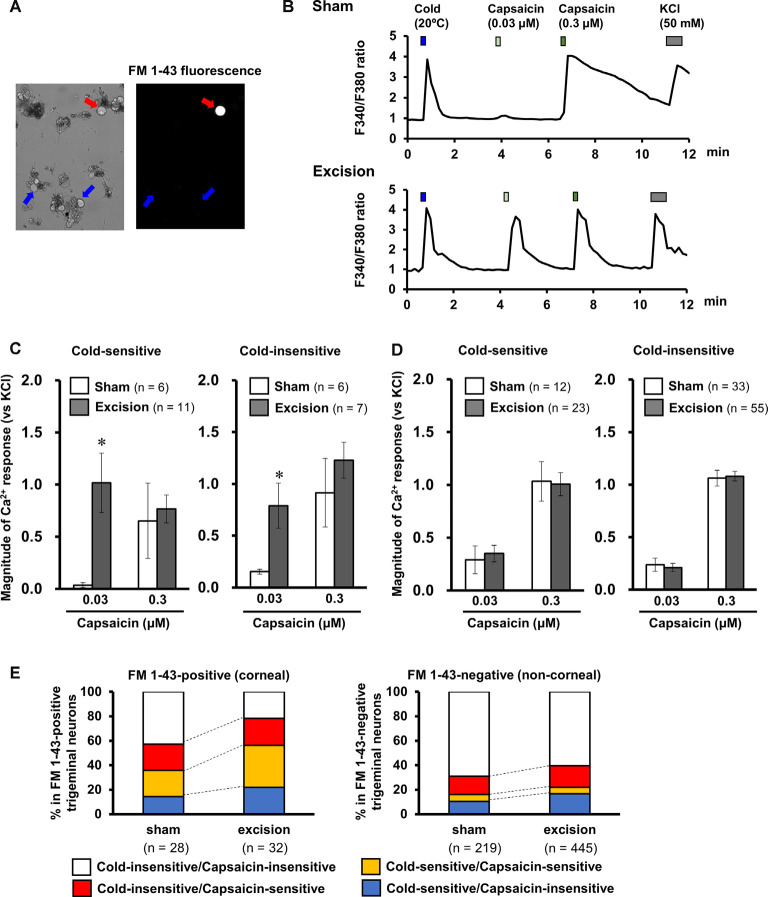
Effect of lacrimal gland excision on capsaicin-induced intracellular calcium response in the corneal and non-corneal trigeminal ganglion neurons. **(A)** Representative image of an FM 1-43-positive corneal neuron (red arrow) and FM 1-43 negative non-corneal neuron (blue arrows). **(B)** Representative trace of the Fura-2 fluorescence ratio on a cold-sensitive neuron labeled by FM1-43 dissociated from the sham-operated (upper) and lacrimal gland excision guinea pigs (bottom). Cold solution, 0.03 and 0.3 μM capsaicin solution, and high potassium solution were applied in order. **(C,D)** The effect of lacrimal gland excision on capsaicin-induced intracellular calcium responses in FM 1-43-positive corneal neurons **(C)** and FM 1-43-negative non-corneal neurons **(D)** divided into cold-sensitive (left) and cold-insensitive neurons (right). The open and gray columns represent the sham-operated and gland excision groups, respectively. **(E)** The changes in the population of cold/capsaicin-sensitive neurons after gland excision in FM 1-43-positive (corneal, left) and FM 1-43-negative neurons (non-corneal, right). Each column and vertical bar represent the mean ± SEM. *P < 0.05 vs. the sham-operated group (two-way ANOVA followed by Dunnett’s test).

Sensitivities to capsaicin were compared in cold-sensitive nerves recorded in the corneas in the sham-operated and gland excision groups. As shown in Figure 3A; capsaicin perfusion for 2 min increased NTI activities in some of the cold-sensitive nerves, while increased NTI activities were not observed during vehicle perfusion (Krebs–Henseleit solution containing 0.1% ethanol, data not shown). The latency time until the increase in NTI activities after capsaicin perfusion was markedly shorter in the gland excision group, although there was no major difference between the groups regarding the magnitude of the response. As can be seen in Figures 3B,C; the response latency significantly decreased in the gland excision group, while no significant difference was found in the total NTI activities in the presence of capsaicin. The short response latency to capsaicin in the gland excision group implies that capsaicin can activate cold-sensitive nerves of the gland excision group at lower concentrations than those of the sham-operated group since the concentration of capsaicin in the recording chamber gradually increased after initiating the perfusion. The lack of change in total NTI activity during capsaicin perfusion suggests that the cold-sensitive nerve excitations by maximal activation of TRPV1 are identical between the sham-operated and the gland excision groups since 1 μM capsaicin can fully activate TRPV1 channels (Masuoka et al., 2015). To further examine whether the capsaicin-induced response was sensitized in the corneal sensory neurons after gland excision, we labeled corneal neurons using the retrograde fluorescence dye FM1-43. We cultured primary trigeminal ganglion neurons from sham-operated and gland-excised guinea pigs. The corneal neurons were identified using FM1-43 fluorescence (Figure 4A), and then the intracellular Ca^2+^ responses induced by cooling (20°C), capsaicin (0.03 and 0.3 μM), and high potassium (KCl) solution were measured with Fura-2 calcium indicator in the FM1-43-positive corneal neurons (Figure 4B) and the neighboring FM1-43-negative non-corneal neurons. Capsaicin-induced Ca^2+^ responses normalized by KCl responses were analyzed in capsaicin-sensitive neurons, in which 0.3 μM capsaicin increased the Ca^2+^ concentration by more than 0.1. Figure 4C shows the magnitude of the Ca^2+^ response to capsaicin in FM1-43-positive corneal neurons divided into cold-sensitive and cold-insensitive neurons. Perfusion of a low concentration of capsaicin (0.03 μM) induced a small or no intracellular Ca^2+^ elevation in the cold-sensitive and cold-insensitive neurons from the sham-operated guinea pigs, whereas a high concentration of capsaicin (0.3 μM) induced a large Ca^2+^ elevation. In the corneal neurons from the gland-excised guinea pigs, capsaicin markedly increased intracellular Ca^2+^ concentration even at a low concentration, regardless of responsiveness to cold. At low concentrations of capsaicin, Ca^2+^ elevations in the FM1-43-positive corneal neurons of the gland excision group were significantly larger than those of the sham-operated group. On the contrary, in the FM1-43-negative non-corneal neurons (Figure 4D), capsaicin-induced intracellular Ca^2+^ elevation showed no significant difference between the sham-operated and gland excision groups. Perfusion of the vehicle alone showed no intracellular calcium response in trigeminal ganglion neurons (data not shown). Therefore, high sensitivity to capsaicin in the corneal NTI response in the gland excision group may be caused by sensitization to TRPV1, while the same total NTI activity induced by 1 μM capsaicin in sham-operated and gland-excised corneas may be related to the same TRPV1 responses induced by a high concentration of capsaicin. Finally, changes in the proportion of neurons that responded to cold and/or capsaicin were analyzed (Figure 4E). The cold-sensitive population in FM 1-43 positive corneal neurons tended to increase after gland excision, although there was no significant difference between the groups (chi-squared test). These results indicate that the TRPV1-mediated response under chronic ocular dryness is sensitized in corneal sensory neurons regardless of cold responsiveness. Sensitization of the TRPV1-mediated response is specific for corneal trigeminal sensory neurons but is not elicited in trigeminal sensory neurons innervating the other tissues.

### Lacrimal Gland Excision Increases the Proportion of Corneal Neurons Enclosed by GFAP-Positive Frames in the Trigeminal Ganglion

**Figure 5:**
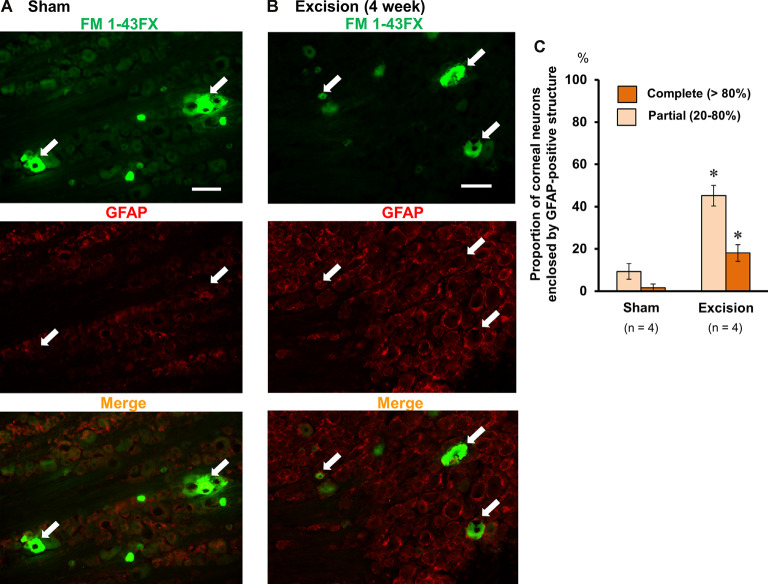
Photomicrographs of FM 1-43FX-positive corneal neurons and glial fibrillary acidic protein (GFAP)-immunoreactive cells in the anteromedial part of the trigeminal ganglion. **(A,B)** The trigeminal ganglion sections of the sham-operated **(A)** and gland excision guinea pigs **(B)**. The corneal neurons were labeled with FM 1-43FX (green in the upper images), and the trigeminal ganglia were subsequently immunostained with anti-GFAP antibody (red in the middle images). The merged FM1-43FX/GFAP images are represented in the lower panels. The white arrows represent the FM 1-43FX-positive corneal neurons. Scale bars = 50 μm. **(C)** Percentage of FM 1-43FX-positive neurons completely (>80%) or partially (20–80%) enclosed by the GFAP-immunoreactive structure in the total FM 1-43FX-positive neurons. *P < 0.05 vs. the sham-operated group (Student’s *t*-test).

Based on a previous report, trigeminal nerve injury is associated with an increase in immunoreactivity for GFAP in satellite glial cells and an increase in trigeminal neurons enclosed by the GFAP-immunopositive structure in the trigeminal ganglia (Stephenson and Byers, 1995), which is linked to phenotypic changes in the trigeminal sensory neurons after nerve injury (Mikuzuki et al., 2017). Therefore, we labeled the corneal neurons with FM 1-43FX dye and made trigeminal ganglion sections followed by immunostaining with an anti-GFAP antibody. The neurons labeled with FM 1-43FX fluorescence (white arrows, upper images in Figures 5A,B) were observed only in the anteromedial part of the trigeminal ganglion. The proportion of FM 1-43FX-positive corneal neurons enclosed by GFAP signals was analyzed in both the sham-operated and gland excision groups. GFAP signals were weak in the sham-operated group. Only 3.7% and 9.3% of the FM 1-43FX-positive neurons were completely and partially surrounded by the GFAP-positive frame, respectively (Figures 5A,C). However, in the gland excision group, the proportion of FM1-43FX-positive neurons enclosed by complete and partial GFAP-positive frames significantly increased to 14.9 and 45.1%, respectively (Figures 5B,C). Therefore, corneal nerve sensitization induced by gland excision may be accompanied by an increase in GFAP expression in satellite glial cells enclosing corneal neurons in the anteromedial part of the trigeminal ganglion.

### Effects of TRPV1 Ligands on Blink Behavior in Sham-Operated and Lacrimal Gland-Excised Guinea Pigs

**Figure 6:**
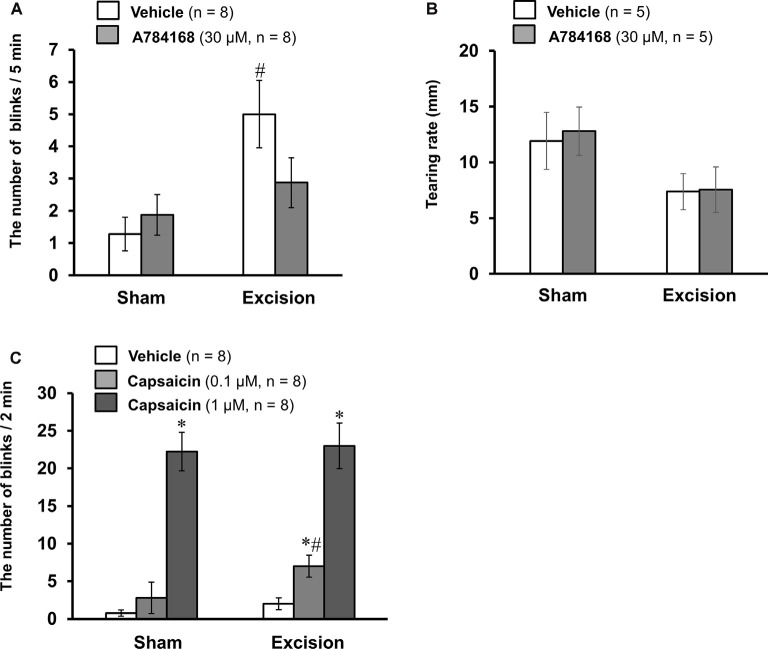
Effects of TRPV1 ligand eye drops on blink behavior in the sham-operated and lacrimal gland-excised guinea pigs. **(A,B)** Effect of the TRPV1 antagonist, A784168, on blink frequency **(A)** and tear volume **(B)**. The number of blinks was counted 1 min after the treatment of the vehicle or 30 μM A784168 for 5 min. Tear volume was measured 5 min after the application of eye drops. **(C)** Effect of the TRPV1 agonist, capsaicin, on blink behavior. The number of blinks was counted for 2 min immediately after the treatment of vehicle or capsaicin (0.1 or 1 μM). Each column and vertical bar represent the mean ± SEM. *P < 0.05 vs. vehicle group, ^#^*P* < 0.05 vs. the sham-operated group (two-way ANOVA followed by Dunnett’s test).

To explore the pathophysiological significance of the sensitization of TRPV1-mediated neuronal responses, we examined the effect of the selective TRPV1 antagonist A784168 on eye blinks. The number of blinks was counted after the topical application of the vehicle or 30 μM A784168 on the ocular surface of the sham-operated and gland-excised guinea pigs (Figure 6A). The gland-excised guinea pigs showed a significantly higher frequency of blinks than sham-operated guinea pigs when the vehicle was used (open columns). However, after topical application of 30 μM A784168, the number of blinks did not increase. The difference in blink frequency between the sham-operated and gland excision groups (gray columns) was not statistically significant. A784168 did not affect tear volume in either the sham-operated or the gland excision group (Figure 6B). The enhancement of eye blinks by lacrimal gland excision may be related to the sensitization of TRPV1-mediated corneal nerve response. Subsequently, capsaicin-induced blink behavior was counted to assess sensitivity to exogenous nociceptive stimuli. A low concentration of capsaicin (0.1 μM) significantly increased the number of blinks in the excision group, but not in the sham-operated group. However, a high concentration of capsaicin (1 μM) induced a large number of eye blinks in both the sham-operated and gland excision groups (Figure 6C). Chronic tear deficiency induced by gland excision is suggested to enhance sensitivity to noxious stimuli *via* TRPV1.

## Discussion

**Figure 7:**
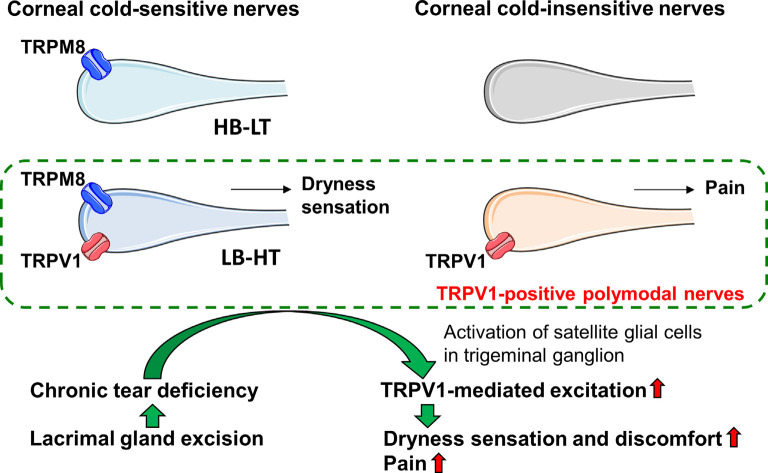
Graphic summary of the main findings. Corneal cold-sensitive nerves with the cold sensor, transient receptor potential melastatin 8 (TRPM8), are classified into high background activity-low threshold (HB-LT) cold thermoreceptor, and low-background activity-high threshold (LB-HT) cold thermoreceptor. The polymodal nociceptor, TRPV1, is expressed in corneal cold-sensitive nerves characterized by LB-HT cold thermoreceptors that provide dryness sensation, as well as in a part of the corneal cold-insensitive nerves that provide pain. Tear deficiency induced by lacrimal gland excision sensitizes the TRPV1-mediated excitation in corneal nerves, possibly mediated by the increase in activated satellite glial cells around the corneal neurons in the trigeminal ganglion. The changes in the sensitivities of corneal nerves might relate to the induction of dryness and discomfort sensation and pain under chronic tear deficiency.

To clarify the contribution of TRPV1 to the corneal nerves resulting in the discomfort experienced in DED, we examined the localization of TRPV1 in corneal cold-sensitive nerves detecting ocular dryness and the changes in TRPV1-mediated neuronal excitation and blink behavior in an experimental model of aqueous-deficient DED. Functional TRPV1 channels expressed in approximately 46–48% of corneal cold-sensitive nerves exhibited low background activity and a high threshold for cooling electrophysiological properties. Chronic tear deficiency induced by lacrimal gland excision sensitized the TRPV1-mediated responses in the corneal cold-sensitive and cold-insensitive neurons, without changing its maximal response. This phenomenon was accompanied by an increase in GFAP-immunopositive satellite glial cells surrounding the corneal neurons in the trigeminal ganglion. TRPV1 antagonist eye drops attenuated the increase in eye blinks after gland excision, whereas blink behavior induced by a low concentration of TRPV1 agonist was potentiated in gland-excised guinea pigs. Therefore, we concluded that chronic tear deficiency induced by lacrimal gland excision sensitizes the TRPV1-mediated response in cold-sensitive and cold-insensitive corneal nerves, which may be related to symptoms of dryness, pain, and hyperalgesia in DED (Figure 7).

Corneal cold-sensitive nerves with the cold sensor, TRPM8, are subdivided into two phenotypes: high background activity-low threshold (HB-LT) cold thermoreceptor and low-background activity-high threshold (LB-HT) cold thermoreceptor (Alcalde et al., 2018; Masuoka et al., 2018). The former, which is responsible for innoxious cooling associated with dryness on the ocular surface, contributes to basal tear secretion, and the latter, which is responsible for noxious cooling and high osmolality associated with severe dryness, is linked to ocular discomfort, irritative tear secretion, and blink response (Belmonte et al., 2015). As shown in Figure 2, the cold-sensitive nerves responsive to the TRPV1 agonist, capsaicin, showed significant low-background activities and tended to have high cooling thresholds. Therefore, the cold-sensitive nerves equipped with TRPV1 may overlap with the LB-HT cold thermoreceptors. TRPV1 is reported to be a typical polymodal nociceptor that can sense heat, acids, and endogenous lipids (Caterina and Julius, 2001). Our findings confirm the hypothesis that the LB-HT cold thermoreceptor contributes to the detection of ocular surface nociception. Also, many LB-HT cold thermoreceptors may be characterized as polymodal sensors that can integrate many types of noxious stimuli. TRPV1 activation may also cause dry eye discomfort through the excitation of LB-HT cold thermoreceptors that detect nociceptive ocular dryness.

Chronic tear deficiency induced by lacrimal gland excision was found to sensitize the TRPV1-mediated response in corneal cold-sensitive neurons (LB-HT cold thermoreceptors) and cold-insensitive neurons. Additionally, our behavioral experiments showed that capsaicin-induced blink behavior was also sensitized in gland-excised guinea pigs (Figure 6C). A similar result was reported in a rat dry eye model 2 weeks after exorbital lacrimal gland excision, which showed an increase in eye wiping behavior and orbicularis oculi muscle activity after treatment with hypertonic saline and capsaicin (Bereiter et al., 2018). We suggest that weak stimuli of TRPV1 below the nociceptive threshold under normal conditions activate LB-HT cold thermoreceptors and cold-insensitive polymodal nociceptors in DED and may result in dry eye discomfort and pain, respectively. We further clarified that the selective TRPV1 antagonist, A784168, ameliorated the increase in eye-blink frequency in chronic tear deficiency (Figure 6A). Ocular surface inflammation, tear film instability, and hyperosmolarity are known to underlie the symptoms of DED (Nelson et al., 2017). Dry eye model mice placed under desiccating conditions for 14 days had a significantly increased expression of cyclooxygenase-2 in the cornea (Li et al., 2010). Furthermore, there are reports that tears from patients with DED contain high concentrations of secretory phospholipase A_2_ (Chen et al., 2009). The abundance of these enzymes could increase arachidonic acid metabolites, such as prostaglandins and leukotrienes, which directly or indirectly activate TRPV1 channels (Hwang et al., 2000; Shin et al., 2002; Moriyama et al., 2005). Ocular surface temperature in the central cornea of patients with DED varies by eye-opening (Kamao et al., 2011). Moreover, an increase in temperature within the physiological range is found to exert a potentiating effect on the response to TRPV1 activators in sensory neurons (Ni and Lee, 2008). The combination of these neurological and pathophysiological changes may exacerbate spontaneous ocular discomfort *via* TRPV1 in DED.

Surprisingly, chronic tear deficiency did not change the ongoing activity and cooling threshold of cold-sensitive nerves. In the same aqueous-deficient dry eye model, chronic ocular dryness decreased potassium current densities and increased sodium current densities, leading to an increase in ongoing activities and the response to cold in cold-sensitive nerves (Kovács et al., 2016). This discrepancy may be caused by the difference in ocular surface conditions that depend on sex and breeding environments, such as humidity, animal density, and cage conditions since many environmental factors affect the severity and pathology of dry eye. Another possibility is the washout effect of endogenous inflammatory mediators during incubation in Krebs–Henseleit solution since corneal recordings were started 1–4 h after the isolation of corneas. Li et al. (2019) claimed that TRPV1 activities induced by endogenous ligands are important for the maintenance of ongoing activities and cold responses in corneal cold-sensitive nerves. The higher activities of corneal cold-sensitive nerves in previous reports would be maintained by endogenous TRPV1 ligands generated by ocular inflammation since perfusion of the TRPV1 antagonist in the present study had no significant effect on ongoing activities in cold-sensitive nerves (data not shown).

Joseph et al. (2019) reported that the phosphorylation of TRPV1 at S801 sensitizes the TRPV1-mediated current response in sensory neurons and potentiates pain behavior, which is involved in the generation of spontaneous pain in masseter inflammation. Therefore, TRPV1 phosphorylation may contribute to the sensitization of the TRPV1-mediated response under chronic tear deficiency in the current study. Changes in the protein expression of TRPV1 are also assumed to be related to sensitization since the sensitization of TRPV1-mediated response in the corneal trigeminal neurons remained after cultivation for a minimum of 1–2 days. Trigeminal neuropathic pain elicited by the inferior alveolar and mental nerve transection was reported to increase TRPV1 expression in the mandibular trigeminal ganglion neurons (Kim et al., 2008). Our study revealed that post-translational modification after an increase in TRPV1 expression is also involved in the sensitization of TRPV1 under inflammation (Masuoka et al., 2020); the expression of TRPV1 and its scaffold protein, A-kinase anchoring protein 5 (AKAP5), is elevated in nociceptive sensory neurons during inflammation, which results in the sensitization of TRPV1 by its persistent phosphorylation. Hence, chronic changes occur in TRPV1 expression, and some proteins that regulate TRPV1 excitation may play a role in the sensitization of the TRPV1-mediated response in corneal nerves in patients with DED.

The sensitization of the TRPV1-mediated response in the corneal nerves was accompanied by an increase in enclosed corneal neurons by GFAP-immunoreactive satellite glial cells in the trigeminal ganglion. Similar phenomena have been reported in many animal models of neuropathic and inflammatory pain. For example, 3–7 days after molar pulp injury, satellite glial cells located around the neuronal somas became immunoreactive for GFAP in the trigeminal ganglion (Stephenson and Byers, 1995). Tooth pump inflammation showed many GFAP-immunoreactive cells and an increase in TRPV1 immunoreactivity in the second branch region of the trigeminal ganglion and simultaneously decreased the threshold to noxious heat stimuli (Watase et al., 2018). Exposure to repetitive tertiary blast resulted in increases in TRPV1, endothelin-1, and GFAP expression in the trigeminal ganglion, and enhanced nociception through a decrease in the desensitization of TRPV1 in the cornea (Por et al., 2017). Donegan et al. (2013) suggested that the enhancement of GFAP expression in the ganglion plays a crucial role in the maintenance of neuropathic pain. GFAP gene knockout or intrathecal administration of GFAP antisense oligodeoxynucleotides extinguished the upregulation of GFAP expression induced by spinal nerve ligation in the ganglion, leading to a reduction in neuropathic pain. Therefore, considering the mechanisms of other neuropathic and inflammatory pain disorders, the chronic sensitization of the TRPV1-mediated response in corneal nociceptive neurons during ocular dryness is possibly related to the neuron-satellite glial cell interaction through GFAP expression in satellite glial cells. However, further experimental studies are necessary to investigate the detailed mechanism of the sensitization of the TRPV1-mediated response.

In conclusion, chronic tear deficiency sensitizes the TRPV1-mediated response in LB-HT cold-sensitive and cold-insensitive polymodal nerves in the cornea, which may serve as therapeutic targets of spontaneous dry eye discomfort and hyperalgesia to nociceptive stimuli in aqueous-deficient dry eyes.

## Data Availability Statement

The raw data supporting the conclusions of this article will be made available by the authors, without undue reservation.

## Ethics Statement

The animal study was reviewed and approved by the Ethics Committee of Kanazawa Medical University.

## Author Contributions

TM designed and performed the research, analyzed the data, and wrote the article. YY, KN, TN, and KT performed the research and analyzed the data. KT, MT, QH, KI, and TI wrote and revised the article. All authors contributed to the article and approved the submitted version.

## Conflict of Interest

The authors declare that the research was conducted in the absence of any commercial or financial relationships that could be construed as a potential conflict of interest.
